# The anti-inflammatory scan: current insights and future perspectives on therapeutic splenic ultrasonography

**DOI:** 10.1186/s13089-025-00446-0

**Published:** 2025-09-22

**Authors:** Pierpaolo Di Nicolò, Francesco Corradi

**Affiliations:** 1 Primary Care Department, AUSL Romagna, Lugo District, Ravenna, Italy; 2 Department of Surgical, Medical, Molecular Pathology and Critical Care Medicine, University of Pisa, Pisa, 56124 Italy; 3 Azienda Ospedaliero Universitaria Pisana, Via Paradisa, 2, Pisa, 56126 Italy

## Abstract

Recent discoveries have identified that physiological anti-inflammatory reflexes are triggered by vagus nerve stimulation (VNS), which offers neuromodulation— performed via implantable or transcutaneous devices—potential therapeutic opportunities. A novel, noninvasive technique using spleen-targeted focused ultrasound stimulation (sFUS) can replicate these effects by triggering the vagus nerve, opening new possibilities for immunomodulation. Early findings suggest that sFUS may evolve into a therapeutic tool to modulate inflammatory responses across a number of diseases. This short communication presents preclinical evidence of efficacy in diverse models of inflammation, discusses the mechanisms underlying sFUS, explores potential translational steps into human application and discusses future directions.

## Introduction

The vagus nerve, composed of approximately 20% efferent and 80% afferent fibers, facilitates bidirectional communication between the brain and peripheral organs, including components of the immune system [1]. The identification of vagus nerve stimulation (VNS)-mediated anti-inflammatory reflexes has established neuromodulation as a promising therapeutic strategy. VNS, typically delivered via implantable or transcutaneous electrical devices, is currently employed in the treatment of epilepsy, depression, and several inflammatory conditions [2–4]. More recently, noninvasive focused ultrasound stimulation of the spleen (sFUS) has been shown to replicate the anti-inflammatory effects of VNS, supporting the development of a novel, image-guided, interventional approach to immune modulation [5].

## Preclinical evidence of anti-inflammatory effects

In rodent models, spleen-focused ultrasound stimulation (sFUS) administered prior to renal or cardiac ischemia–reperfusion injury significantly reduces renal tubulointerstitial fibrosis—by up to 90%—and decreases myocardial infarct size by nearly threefold [6, 7]. In experimental models of inflammatory arthritis in mice, sFUS demonstrates both prophylactic and therapeutic effects, mitigating disease severity and preventing disease progression [8]. In sepsis models, it has been shown to suppress proinflammatory cytokine release [9]. These findings across diverse disease states have thus prompted further investigation into the underlying physical and biological mechanisms through which ultrasound may mediate immunomodulation.

## Mechanisms of action

**Fig. 1:**
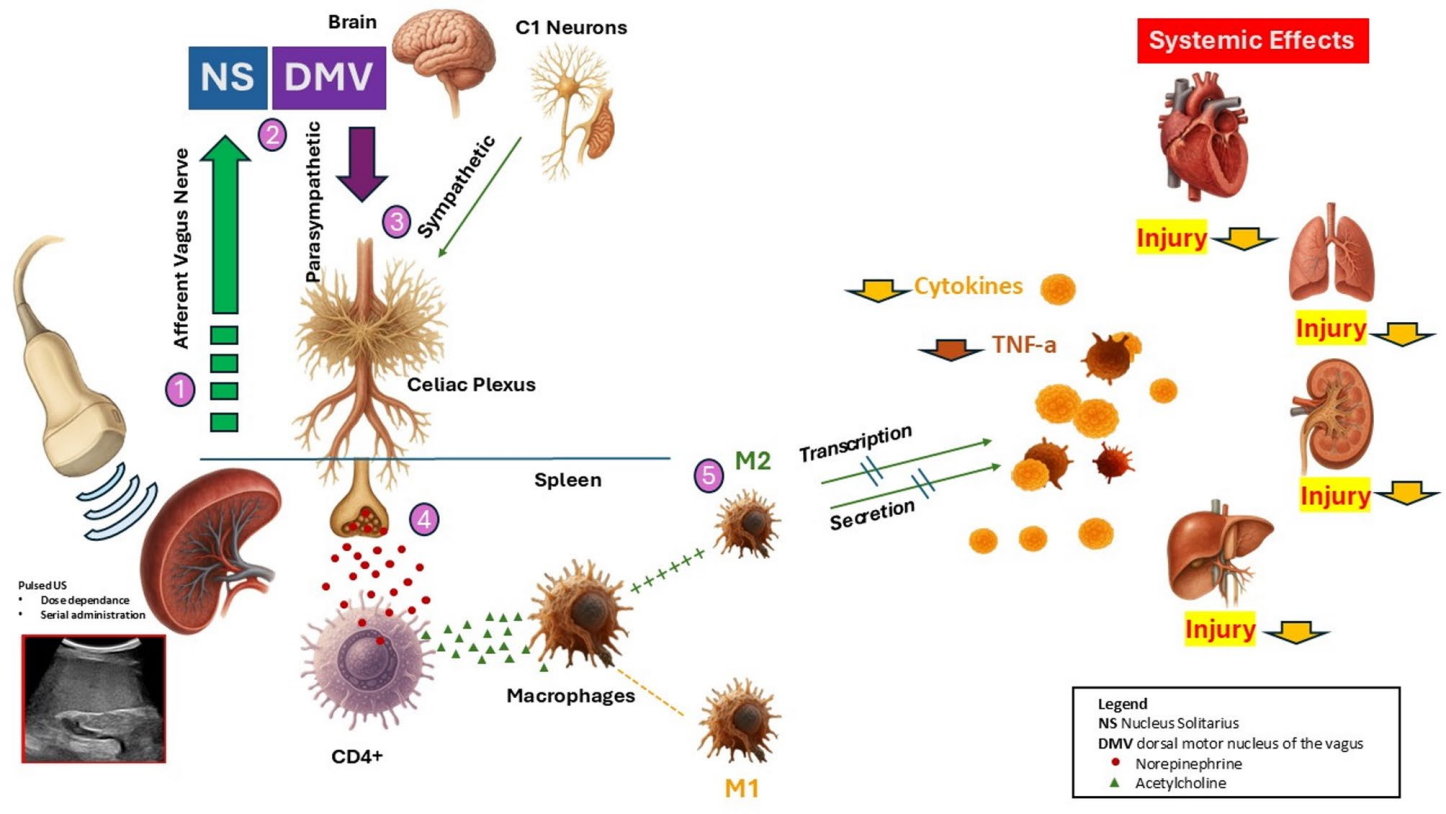
Currently postulated mechanisms of neuroimmune modulation in the spleen. (**1**) Vagus nerve stimulation with pulsed ultrasound activates the cholinergic anti-inflammatory pathway (CAP) via the vagus nerve to the Nucleus solitarius (NS). (**2**) The NS connects to C1efferent sympathetic neurons as well as the dorsal motor nucleus of the Vagus (DMV) in the brainstem. From there (**3**) sympathetic and parasympathetic efferent fibers travel to the celiac plexus, synapsing with postganglionic efferent fibers projecting to the spleen. In the spleen (**4**), the efferent nerves release norepinephrine, activating adrenergic receptors on CD4 + T cells. The subsequent production of acetylcholine (ACh), which binds to nicotinic receptors on splenic macrophages leads to increased production of the M2-like (organ-protective) phenotype that inhibit release of proinflammatory cytokines (**5**), ultimately affecting immune responses in end organs throughout the body. Acute stimulus of the reflex appears to inhibit release of cytokines such as tumor necrosis factor alfa (TNF-a) while chronic stimulation also appears to inhibit their transcription. Organ protective and anti-inflammatory effects of the described reflex are dependent on intact splenic nerve-to-macrophage signaling

sFUS generates alternating zones of high and low pressure within splenic tissue, inducing a range of physical effects, including localized thermal changes, mechanical wave propagation, cavitation, and viscosity-related fluid dynamics [5, 10]. Although the downstream signaling pathways remain incompletely characterized, a key proposed mechanism involves activation of the cholinergic anti-inflammatory pathway (CAP) [Fig. 1]. CAP is a pathway that regulates the inflammatory response to stimuli by inhibiting the release of pro-inflammatory cytokines.

Ultrasound stimulation of splenic afferent vagal fibers can activate CAP by initiating signaling to the nucleus tractus solitarius in the medulla. This central relay projects to sympathetic nuclei and vagal efferents targeting the celiac plexus, which subsequently activates postganglionic neurons innervating the spleen. These neurons release norepinephrine, which stimulates splenic CD4⁺ T cells to produce ACh. ACh, in turn, binds to macrophage acetylcholine receptors, suppressing TNF-a production and promoting an M2-like anti-inflammatory phenotype—a cascade known as the CAP [6, 11, 12].

Conceptually, the CAP can be described as a neuroimmune interface occurring within the spleen, mediated by crosstalk between the vagus nerve and CD4⁺ T cells. This pathway is disrupted in animals lacking a spleen, T cells, or macrophage acetylcholine receptors, underscoring the critical importance of intact splenic nerve–immune cell signaling for CAP function [13].

Preclinical studies show that CAP activation by sFUS is dose-dependent, with longer and repeated stimulations yielding stronger and longer-lasting anti-inflammatory responses [8, 11]. Acute sFUS suppresses TNF-a protein release, while chronic exposure modulates gene transcription, decreasing TNF-a mRNA expression [14]. This transcriptional modulation is also implicated in animal models of pulmonary arterial hypertension, where sFUS reduces lung inflammation, right ventricular pressure, and hypertrophy [15].

## Early explorations in therapeutic applications in humans

Therapeutic applications of sFUS in humans remained speculative until 2023, when trials demonstrated activation of the anti-inflammatory reflex using diagnostic-level ultrasound energy in humans. In these studies, sFUS applied to either the splenic hilum or parenchyma resulted in a 4 to 5 fold reduction in TNF-a production by white blood cells following ex vivo endotoxin stimulation [16]. No adverse clinical, biochemical, or hematological effects were observed. TNF-a levels in these studies returned to baseline within 24 h, suggesting that repeated insonation may be necessary for the management of chronic inflammatory conditions, although the exact dose and frequency requirements remain unknown [16].

## Clinical implications and future directions

Ongoing pilot trials are currently evaluating the therapeutic potential of sFUS in chronic inflammatory diseases and acute sepsis [17, 18]. The growing recognition of perivascular inflammatory signaling in acute kidney injury (AKI) [19] further supports the potential utility of sFUS in perioperative care and in contexts requiring contrast, such as the prevention of contrast-induced nephropathy. Future investigations should explore whether sFUS can attenuate glomerular diseases, reduce the risk of allograft rejection, or provide benefit in other immune-mediated conditions.

## Conclusion

Though still in its early stages, the concept of an “anti-inflammatory scan” through sFUS represents a potentially transformative paradigm in non-invasive immunomodulation, but warrants rigorous study and exploration. It is time to assess whether an “anti-inflammatory scan” via sFUS neuromodulation can be a meaningful addition to our therapeutic armamentarium where imaging and intervention converge in a single pulse.

## Data Availability

Data sharing is not applicable to this article as no datasets were generated or analysed.
